# Disadvantaged populations in maternal health in China who and why?

**DOI:** 10.3402/gha.v6i0.19542

**Published:** 2013-04-03

**Authors:** Beibei Yuan, Xu Qian, Sarah Thomsen

**Affiliations:** 1 Department of Public Health, Division of Global Health (IHCAR), Karolinska Institutet, Solna, Sweden; 2 China Center for Health Development Studies, Peking University, Beijing, China; 3 School of Public Health, Fudan University, Shanghai, China

**Keywords:** maternal health, equity, disadvantaged populations, China

## Abstract

**Background:**

China has made impressive progress towards the Millennium Development Goal (MDG) for maternal and reproductive health, but ensuring that progress reaches all segments of the population remains a challenge for policy makers. The aim of this review is to map disadvantaged populations in terms of maternal health in China, and to explain the causes of these inequities to promote policy action.

**Methods:**

We searched PUBMED, Popline, Proquest and WanFang and included primary studies conducted in mainland China. Experts were also contacted to identify additional studies. Disadvantaged populations in terms of MDG 5 and the reasons for this disadvantage explored by authors were identified and coded based on the conceptual framework developed by the WHO Commission on the Social Determinants of Health.

**Results:**

In China, differences in maternal health service utilization and the maternal mortality ratio among different income groups, and among regions with different socio-economic development still exist, although these differences are narrowing. Groups with low levels of education and ethnic minorities utilize maternal health care less frequently and experience higher maternal mortality, although we could not determine whether these differences have changed in the last decade. Rural-to-urban migrants use maternal health care and contraception to a lower extent than permanent residents of cities, and differential maternal mortality shows a widening trend among these groups. Gender inequity also contributes to the disadvantaged position of women. Intermediary factors that explain these inequities include material circumstances such as long distances to health facilities for women living in remote areas, behavioral factors such as traditional beliefs that result in reduced care seeking among ethnic minorities, and health system determinants such as out-of-pocket payments posing financial barriers for the poor.

**Conclusions:**

Inequity in maternal health continues to be an issue worthy of greater programmatic and monitoring efforts in China.

Previous studies suggest general progress towards improved maternal health throughout the world (1, 2). However, a growing body of evidence shows that this progress is failing to reach the most disadvantaged populations (1, 3, 4). This evidence also confirms that reducing health inequities and improving maternal health in disadvantaged populations is crucial in order to meet the fifth Millennium Development Goal in a universal manner.

China has made impressive progress in maternal health nationally. The overall maternal mortality ratio decreased from 64/100 000 in 1996 to 38/100 000 in 2008 (5); overall hospital births increased to 94.7% of all live births in 2008 (6); and overall contraceptive use was above 80% in 2006 (7). These achievements can be attributed to China's rapid economic development and the efforts of the government to promote maternal health services (8–10). In 1994, China issued the Law on Maternal and Infant Health care. With the national laws and some auxiliary regulations, the legal system has been established to protect the health of women and children in China in the 1990s (8). China also established a comprehensive and multilevel maternal health services delivery system covering urban and rural areas. Since the 1990s, the Chinese government and some international organizations have implemented a number of programs to promote maternal health especially in poor rural or remote areas. Some examples are Strengthening MCH and Family Planning Services in Grassroots of China (UNICEF/UNFPA/WHO, 1990–2000), the MCH and AIDS Control Project (World Bank Loan, 1999–2008), and Reducing Maternal Mortality and Eliminating Neonatal Tetanus (Ministry of Health, State Council's National Working Committee for Children and Women and Ministry of Finance, 2002–2006). The interventions in these programs included improving maternal health facilities in these areas, training staff, or directly providing institutional delivery subsidies to local residents (8, 10). In 2009, the Health Care System Reform established the target of Equalization of Basic Public Health Services. As the core content of basic public health services, some relevant projects on maternal health care have been implemented, including folic acid supplement for rural women before pregnancy and in the first trimester and subsidies for institutional delivery (11).

Despite these considerable efforts, analyses of sub-groups of populations in China in recent decades shows that there are still large sectors of the population that are disadvantaged in regards to maternal health. Disparities in maternal health service utilization between low- and high-income households still exist (6). The status of maternal health in the poorest rural and remote areas remains very poor (5, 6), and rural women still receive less antenatal and post-partum care than urban women (10). Ethnic minorities in China face geographic marginalisation and poor quality health facilities in the remote areas where they live; as a result they continue to have a high rate of home births and maternal mortality (12, 13). In recent decades the form of China's economic development has resulted in a great number of rural-to-urban migrants, who are shown to use fewer maternal health care services and contraception, and who have higher maternal mortality than permanent residents of urban areas (14–16). Moreover, because of restrictions on the number of births and son preference in Chinese culture, unauthorized pregnancies and having achieved a son both reduced the likelihood of women accessing maternity care (17, 18).

Health inequity is unfair and should be avoidable or remediable by policies (19, 20). In order to reduce inequity in maternal health, it is necessary to identify the disadvantaged populations in maternal health, to map inequities in these populations to observe if these inequities are worsening or improving, and to analyze the determinants and causes of these inequities.

The aim of this review was to map out the current situation of maternal health with respect to disadvantaged populations in China and the causes of the inequity applying the social determinants of health inequities framework; and propose suggestions for policy and future studies. The specific questions to be answered were:

1. Who are the disadvantaged populations in China and where do they live?
2. How is the inequitable distribution of health in China explained in terms of the social determinants of a health model?

## Methods

### Theoretical framework for health inequity analysis

A conceptual framework of the social determinants of health inequities was developed by the WHO Commission on Social Determinants of Health (20). It provides an opportunity to better understand how inequities in health are rooted in societal structures. It was developed to enhance understanding of the underlying mechanisms of health inequities. It not only places the issue of health inequities in a social context, it also illustrates entry points for intervention and policy. The framework divides the social determinants into structural and intermediary determinants. The structural determinants include context, structural mechanisms such as gender and cultural norms and the resultant socio-economic position, such as income, ethnicity and education. The structural determinants of health inequities operate and affect health through so called intermediary determinants. These include material circumstances such as housing quality and physical environment, psychosocial circumstances such as stressful living circumstances and relationships, (lack of) social support and coping styles, and behavioral and biological factors such as lifestyle and genetic factors. The health system is also described as a social determinant of health, particularly since it mediates the differential consequences of ill health.

### Criteria for considering studies for this review

#### Types of studies

We included studies describing the maternal health situation in disadvantaged populations, or studies comparing the maternal health situation between disadvantaged populations and other populations, or studies analyzing the causes or determinants of the inequity in any of Millennium Development Goal (MDG) 5 goals. We only included studies from mainland China. Cross sectional studies, longitudinal studies or qualitative studies could all be included in our literature review, but review articles were excluded.

#### Types of disadvantaged population

A disadvantaged population is defined as any group in a society that is denied benefits or services because of ‘irrelevant’ characteristics, such as income, race, gender, religion, ethnicity, caste, geography, or residence (e.g. rural/urban/urban slum) (21). Based on this definition, and after consulting experts on maternal health in China, the disadvantaged populations in China that we searched for and included in this literature review are low-income populations, ethnic minorities, populations living in rural or remote areas, rural-to-urban migrants and the low-educated populations.

#### Types of maternal health outcomes

We included articles or reports studying any of the official MDG 5 indicators in disadvantaged populations, which are maternal mortality, births attended by skilled health personnel, antenatal care use, contraceptive use, adolescent birth and unmet need for family planning. Some studies did not research MDG 5 indicators as their independent variable, but researched some intermediary factors in the CSDH framework as independent variables like nutritional status of pregnant women, knowledge of maternal health or attitude towards contraceptives. These studies were also included in the review.

### Search methods

We searched four electronic databases (PubMed, Popline, Proquest Dissertation & Theses Database, Wanfang Dissertation Database) for literature published in English or Chinese after 2000. The PubMed search strategy includes MESH terms and free-text terms for maternal health, disadvantaged populations and China. The search strategy in the other three databases only included the free-text terms. We also contacted experts in the area of maternal health to identify additional articles, PhD dissertations, or reports.

### Selection of studies

The selection process was carried out by the first author and discussed with other authors. The articles had to meet specific inclusion criteria in order to be considered for analysis. In brief, the inclusion criteria were: 1) the article had to be related to any indicators of MDG 5; 2) the article should address one or more disadvantaged groups we defined or factors that make people vulnerable; and 3) articles should have been published between 2000 and the present.

### Data extraction and analysis

**Fig. 1:**
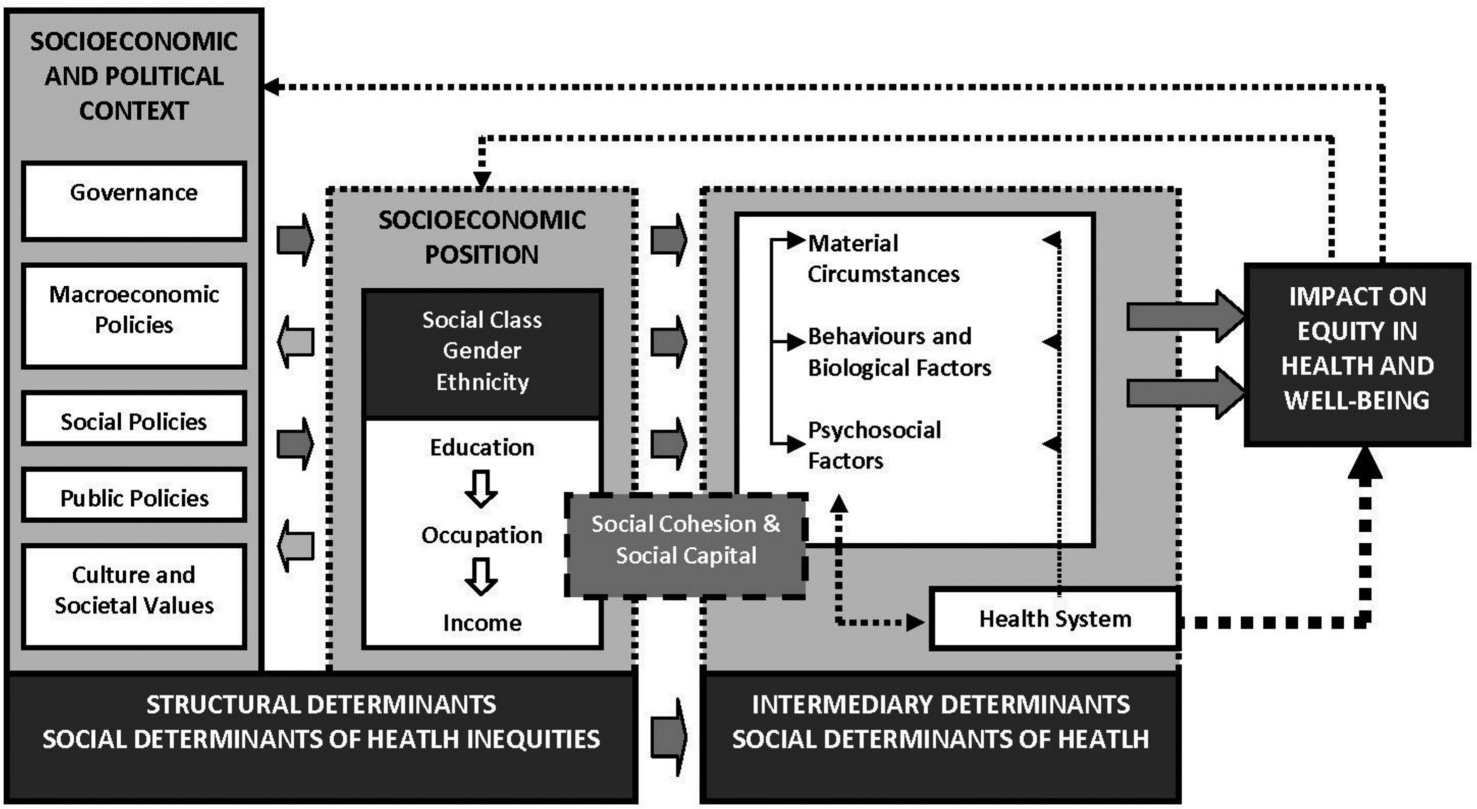
Social determinants of health framework. From ref. 10.

Included articles were analyzed qualitatively with an amended form of content analysis. The theoretical framework of the social determinants for health equity (CSDH, Fig. 1) was used for analysis. The outcomes and discussion content in the literature were thus extracted and grouped according to the type of determinants presented in the CSDH document, which were: 1) socio-economic and political context – governance, macroeconomic policies, social policies, public policies, and cultural and societal values; 2) Structural determinants and socio-economic position (the disadvantaged groups) – economic status, place of residence, education, ethnicity, migrant status, gender; and 3) Intermediary determinants – material circumstances, behaviors and biological factors, psychosocial factors, the health system. Based on the structural determents in CSDH framework, we firstly classified the disadvantaged populations in maternal health identified from the literature and summarized the recent inequity situation of these disadvantaged populations in MDG 5 extracted from included studies. The causes of inequity found or discussed by the article authors are then categorized into one of the four intermediary determinants and synthesized.

The methodological quality of the included studies was assessed with a list of quality criteria developed by Say (22) in her systematic review for observational studies. Qualitative studies were assessed by Critical Appraisal Skills Programme criteria (23). The quality of studies was assessed by the first author and checked by the last author.

## Results

**Fig. 2:**
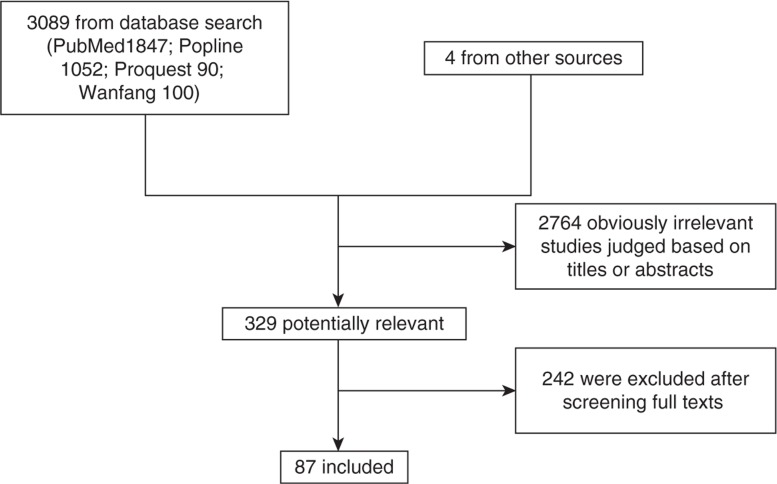
Study selection process.

We retrieved 329 potentially relevant papers on maternal health of disadvantaged population in China after screening 3,093 titles and abstracts (after eliminating duplicates between the three databases). We then examined the full texts of these 329 articles. Finally, 87 closely relevant papers were left and included for data extraction and analysis (Fig. 2). Seventy-two (82.8%) were published after 2005; fifteen (17.2%) were published between 2001 and 2005. All included studies were cross-sectional studies, repeated cross-sectional studies, case control studies or qualitative studies. Most of the included studies were well-conducted with moderate quality based on the assessment criteria (Characteristics and quality assessment of all included studies published as a supplementary material).

### Economic status and place of residence

Low economic status is related to higher maternal mortality or lower utilization of antenatal care or institutional delivery. Economic status is the main structural determinant for health inequity in China according to the identified literature, and was explored by nearly half of the included studies (Table 1). In these studies, individual economic status was measured in different ways, including individual/household income, wealth index, asset situation and bank deposits. In 2008, the National Health Services Survey indicated that the gap in institutional delivery rate between low- and high-income households still existed (96.1% and 87.7% of high- and low-income households, respectively) (6). Data from western provinces of China in 2007 showed that women in the high-income group were 1.7 times more likely to attend at least five pre-natal care visits than those from low-income groups (24).

**Table 1:** The structural determinants of inequity in maternal health

	MMR	SBA	ANC	CON	AB	UNFP	Total
Economic status	3	15	18	2	–	–	38
Place of residence							
Urban/rural areas	8	5	7	–	–	–	20
Different socio-economic development regions	4	5	4	–	–	–	13
Different geographic regions	3	3	6	–	–	–	12
Education	3	21	25	6	–	1	56
Ethnicity	2	10	12	–	–	–	24
Migrant status	4	7	8	12	–	2	33
Gender	–	9	7	4	–	–	20
Occupation	–	4	3	3	–	1	11

The number is the times of each structural determinant were researched in included studies, and one article may study or discuss more than one kind of structural determinant.

MMR: maternal mortality ratio; SBA: skilled birth attendance; ANC: antenatal care; CON: contraceptive use; AB: adolescent birth; UNFP: unmet need for family planning.

Many of the included studies in China analyzed inequity in maternal mortality and maternal health services utilization between rural and urban areas and among regions with different socio-economic development levels. We refer to this here as ‘place of residence.’ Inequities between places of residence have been shown to be closely correlated to economic status, ethnicity and educational level in China, so ‘place of residence’ is included here under economic status (9, 25). Because of the rural–urban dual structure and the unbalanced economic development in China, the populations in rural, inland, and western areas have a disproportionately low economic status compared with the rest of the country. A study using data from the National Maternal and Child Mortality Surveillance System (MCMS) showed that the maternal mortality ratio (MMR) in the poorest rural areas was almost 5 times higher than in urban areas in 2006 (5), and the MMR was highest in remote areas followed by inlands and coastal regions (9).

The good news is that inequities in maternal health based on socio-economic status or regional variations in socio-economic development seem to be reducing in the last two decades in China. We found that eight longitudinal studies (5, 6, 9, 10, 24, 26–28) observed a narrowing of differences in MMR, utilization of pre-natal care and institutional delivery among individuals in different income groups, between rural and urban areas, as well as between remote, inland and coastal regions.

We did not find any studies exploring the economic status in relation to adolescent birth rate and unmet need for family planning in China.

#### Material circumstances

Low economic status was directly related to poor physical conditions of houses or communities, e.g. poor sanitation, no clean drinking water or lack of vehicles (29, 30). It has been shown that rural pregnant women consumed less supplements and nutritious food than those from urban areas (31). In addition, in the remote areas where more low-income populations reside, long distance to health facilities combined with poor transportation deter those populations to access maternal health services (6, 32, 33).

#### Behaviors and biological factors

Women with low economic status or those living in less developed areas had insufficient knowledge about contraceptive methods or the importance of maternal health care, which also contributed to less maternal health care seeking behavior among these poor women (27, 30).

Fines and penalties for not complying with regulations on family size in some areas has been proven to be a deterrent to seeking pre-natal care and institutional delivery for those women who are giving birth to the second or third child in China (17, 18, 34). Low income or rural families usually have more children (17, 35), so they are more likely to reduce their health care seeking behavior in order to temporarily escape the financial penalties.

#### Psychosocial factors

No studies about the impact of low economic status on psychosocial problems related to maternal health in China were found.

#### The health system

In China, the main source of financing the health system is individuals’ out-of-pocket payments. Out-of-pocket payments for pre-natal care and institutional delivery represent a financial barrier for very poor populations to access maternal health care (10, 36). In recent years, the New Rural Cooperative Medical Scheme (NCMS) covering institutional delivery for enrolled rural residents and some governmental or international programs targeting improving maternal health in remote and rural areas have reduced the financial burden of maternal health care on households (24, 37). However, pre-natal care is still seldom covered by the NCMS (24), and the limited reimbursement by NCMS (38, 39) or subsidies by governmental or international programs have not been successful in removing the financial burden on the poorest households.

It was also found that the township health centers or village clinics in poor or remote areas usually have poor equipment and shortages of qualified health workers with insufficient public funding for the health provision system (8, 27, 40). The poor quality of maternal health services, including negative staff attitudes, provided by these health facilities prevents the poor or rural populations from seeking maternal health care (6, 36, 41) and has resulted in higher maternal mortality in poor or remote areas (9, 27, 42). Doctors’ judgmental attitudes to those seeking contraceptive or reproductive health consultations were also found be barriers of reproductive health service utilization (43).

### Education level

Educational level also emerged as an important determinant of maternal health in more than half of our included studies. The National Health Services Survey data from 2008 showed that women with a college education or higher were 2.86 times more likely to give birth in a hospital than those who were illiterate (6). A study in two provinces in 2007 found that women with highschool or higher education were 3.19 times more likely to receive any professional prenatal care than those with elementary education or less (44). The relationship between higher education and contraceptive use was also shown by studies in Anhui and Shanghai (45, 46). It was also shown that women with less than 7 years’ education were 1.49 times more likely to have an unmet need for contraception than those with education of more than 7 years in 2008 (47). Analyses on the district level also found that illiteracy was significantly associated with increased MMR (30, 42). Education is sometimes included in composite measures of the socio-economic development level of a region in China (5, 6, 10), so studies comparing the MMR or maternal health services utilization among rural and urban areas with different socio-economic development levels could also illustrate the influence of education on maternal health. We did not find any studies analyzing the impact of education level on adolescent birth rate in China.

Two longitudinal studies found that inequities in prenatal care and institutional delivery between educational levels had not improved in the 1990s (26, 33), but there have been no studies exploring if these differences are narrowing or widening after 2000.

#### Material circumstances

No studies indicating that educational level results in women's poor living or working environments were found.

#### Behaviors and biological factors

Several included studies showed that a low educational level (less than 6 years or illiterate) is closely related to limited knowledge about maternal health, contraceptives and warning signs of complications during pregnancy (15, 16), which, in turn, hinders poorly educated women from seeking maternal health care and using contraception (44, 46, 48).

#### Psychosocial factors

No studies about the impact of low education status on psychosocial problem related to maternal health in China were found.

#### The health system

Theoretically, educated women are more receptive to health information and benefits of maternal health care, which enables them to better communicate with health providers and thus access maternal health services. This relationship was discussed in two included studies though it was not empirically analyzed (16, 30).

### Ethnicity

There are 56 different ethnic groups in China, of which Han is the majority group, constituting 90.56% of the population. Ethnic minorities are disadvantaged in maternal health. Studies in the western provinces of China showed that ethnic minority women had lower utilization of maternal health care compared with Han ethnicity in the last 5 years, including at least five pre-natal visits, health facility delivery (13, 24, 44). We did not find any studies analyzing the MMR of minority groups per se in the last 10 years. However, ethnic minorities mainly reside in the western or remote provinces of China (30), so the studies showing differences in MMR between remote and coastal regions could also represent the disadvantaged position of ethnic minorities (9, 27). For example, the MMR in Xinjiang Uigur autonomous region, where ethnic minority groups account for approximately 60% of the population, was 3.3 times higher than the MMR in China in 2004 (12).

We did not find any studies analyzing the adolescent birth rate or an unmet need for family planning of ethnic minorities in China.

#### Material circumstances

Because the majority of ethnic minorities in China live in remote, mountainous or nomadic areas, they usually face poor living and transportation conditions, and long distances to health facilities (12, 30, 49, 50), all of which are detrimental to their health status and their accessibility to qualified maternal health care.

#### Behaviors and biological factors

Several studies have shown that minority women lacked basic knowledge on risk factors during pregnancy, and the necessity of facility-based delivery, pre-natal examinations or using contraceptives (28, 51). In addition, cultural preferences for home delivery were found in several ethnic minorities like Hui, Uigur and some ethnic minorities in Sichuan Province (12, 50). For example, women from some ethnic minorities in Xinjiang Province should not been seen by strangers until 12 days after delivery based their cultural belief (12). Together, limited knowledge and traditional beliefs seem to result in ethnic minorities being less likely to seek maternal health care.

#### Psychosocial factors

No studies found.

#### The health system

With limited public subsidies, township health centers cannot provide qualified and culturally-acceptable maternal health care to ethnic minorities (27, 50), such as privacy in the hospital environment, female health workers and option to be accompanied by relatives. One interview study also found that some modern delivery procedures (like pubic shaving) made ethnic minority women feel uncomfortable and embarrassed (6, 50).

The studies showed that out-of-pocket payments also represent a financial barrier for ethnic minorities to access pre-natal care and institutional delivery since women from ethnic minorities usually have low economic status (12, 50). At the same time, a study in China from 2006 found that even if the delivery was free, 10–20% of women still refused to deliver in hospitals (52), so out-of-pocket payments may not be the fundamental cause for minorities using less maternal health services.

### Rural-to-urban migrants

With economic development and industrialization in China, a large proportion of rural laborers (estimated to be 221 million in 2010 (53)) migrated to provincial capital cities or developed coastal cities (i.e. Beijing, Shanghai, Shenzhen) to find a job in the last two decades. Rural-to-urban migrants have become a disadvantaged population in maternal health compared with permanent residents in these cities. In 2008 the MMR of migrants in Zhejiang province (a developed province in coastal China) was 21.67 per 100,000, which was more than three times higher than the MMR of permanent residents of this province (6.57) (29). In Shanghai, the MMR of migrants was nearly 50 times higher than MMR in permanent residents in 2005 (48.46 compared to 1.64) (16). Longitudinal studies from the 1990s to 2005 did not observe any narrowing in the difference of MMR between migrants and permanent residents in Beijing, Shanghai and Zhejiang (16, 28, 54). It has also been shown that rural-to-urban migrants had lower contraceptive use and a higher unmet need for modern contraception than permanent residents (14, 47, 55–59), resulting in induced abortions in small private clinics for unmarried migrants (14, 60).

#### Material circumstances

Rural-to-urban migrants rarely have a professional education background, and are thus more likely to do low-paying manual work in construction, small non-state run factories or commercial service sectors (14, 28). Furthermore, without official household registration in cities, they are not entitled to most public-funded programs, such as housing, education and health services (15, 16). The result is that migrants usually have a vulnerable standard of living and poor working environments and conditions. The low-income status also puts constraints on being able to afford health services (which they must pay for themselves) and other goods or services.

#### Behaviors and biological factors

Migrant knowledge about pregnancy, fertilization and contraception has been found to be very low (55, 61), as is knowledge on the importance of maternal health services (15). Together with economic considerations, poor knowledge and attitudes contributes to their low utilization of contraceptives and maternal health services (15, 61).

Young migrants who travel to urban areas without their parents tend to behave less conservatively and lack knowledge on contraception, so they are more likely to have unprotected, premarital sex than if they lived with their parents, resulting in a higher risk of unwanted pregnancy and abortion among young migrants (14, 60).

#### Psychosocial factors

Migrants may have higher psychosocial stress when they encounter critical life events such as childbirth because they are not familiar with the new urban environments, lack social support networks, and are unaware of procedures and instructions in big, urban hospitals (16).

#### The health system

One qualitative study showed some health system factors contributed to less contraceptive use and more unsafe abortions among migrants: young migrants felt their privacy in reproductive health and abortion consultations was less protected in public health facilities; and reported that they do not use official family planning services because they do not advise their services as much as private clinics run by uncertified practitioners (14).

### Gender

Cultural traditions in China dictate the dominant role of men in the family and strong son preference, especially in rural areas. It has been reported that gender equity awareness between husband and wife was positively related to utilization of pre-natal care and institutional delivery (44, 62), while male spouse's disapproval was an important barrier of contraceptive use (14, 46, 48). Studies have also shown that women who have had a son were less likely to obtain pre-natal care or institutional delivery (17, 63).

#### Material circumstances

Studies have shown that men still hold the power when it comes to financial decision-making in most rural families. In more than half of the rural families studied, women had to get the approval of her husband when spending money on daily necessities and expensive items (64, 65), which may potentially influence women's consumption of health services. However, rural women with experience working in cities were associated with greater odds of using maternal health care compared with those without such experience (35, 66).

#### Behaviors and biological factors

Gender inequity is reflected in the division of labor work within families: in rural areas women usually have to undertake both housing and farming work, which leaves them with little time for personal hygiene and maternal health care (66). Furthermore, most rural women still have relatively poor awareness of gender equity. For example, approximately 60% of women interviewed in a study agreed that ‘the husband's health is more important than the wife's in the family’ (64), which may affect health-seeking behavior.

Lack of control over choosing maternal or reproductive health care could also mirror gender inequity in China. It has also been shown that in some rural areas with more ethnic minorities, women cannot decide where their delivery will take place; this decision is made by their mother-in-law and husband (12). Males were also found to play a key role in choosing the appropriate contraceptive methods (55).

The preference for sons in China results in women who have had a son being less likely to be cared for by themselves or their families during their next pregnancy. One study showed that already having a son reduced the chances that mothers stop heavy physical work before birth during a subsequent pregnancy (66), and women without an existing son were more likely to attend pre-natal care and institutional delivery (17, 63). Given that the Chinese family planning policy controls the number of children (one or two for one couple), greater use of pre-natal care services for women without sons maybe also be attributed to the fact that a pre-natal examination can be used to identify the sex of the fetus with a view to sex-selective abortion if necessary. This can seen in findings that in some areas, sex ratios at birth are higher for second and higher-order births to mothers who made use of prenatal care and skilled birth attendance than to those who did not use such services (17).

#### Psychosocial factors

No studies found.

#### The health system

We did not find any studies analyzing how characteristics of the health system influenced gender inequity related to maternal health. However, lack of privacy during service provision and the lack of female doctors, as discussed above, undoubtedly deter women from accessing maternal or reproductive health care (14, 50, 67).

## Discussion

By making use of the CSDH theoretical framework, we have summarized those who are disadvantaged in maternal health in China and have categorized the causes that generate these inequities in Table 2.

**Table 2:** Structural and intermediary determinants of inequity in maternal health in China

	Economic status and place of residence	Education level	Ethnicity	Rural-to-urban migrants	Gender
Material circumstances	Poor sanitation Lack of vehicles Less supplements and nutritious food Long distance to health facilities	No studies found	Poor living and transportation conditions Long distance to health facilities	Low-paying work Vulnerable living status Poor working environments	Lack of power on financial decision-making in families
Behaviors and biological factors	Insufficient knowledge on importance of maternal health care Insufficient knowledge on contraceptives More children and fine for not complying with family planning policy	Limited knowledge on maternal health and contraceptives	Insufficient knowledge on importance of maternal health care Insufficient knowledge on contraceptives Preference on home delivery	Limited knowledge about contraceptives and importance of maternal health care Less conservatively behaviors	Undertaking both housing and farming work Poor awareness on gender equity Lock control on using maternal health services or contraceptives Son preference and sex-selection abortion
Psychosocial factors	No studies found	No studies found	No studies found	Being not familiar with new environments Lack of social support networks	No studies found
Health system factors	Out-of-pocket payment Insurance Status Coverage of subsidy programs Low quality of maternal health care provided by local health facilities Health workers’ attitude	Poor communication with health workers	Lack of privacy in health facilities Lack of female health workers Lack of cultural-acceptable maternal health services procedures Out-of-pocket payment	Lack of privacy in reproductive health and abortion consultation in public health facilities	Lack of privacy in health facilities Lack of female health workers

### Structural determinants

In China, the differences in maternal health service utilization and MMR among different income groups, between rural and urban areas and among regions with different socio-economic development still exist, although these differences are narrowing, likely due to subsidy programs targeting rural and poor areas and coverage of maternal health care by NCMS. However, women in the poorest families or living in the most remote regions have been left behind.

Educational level was most frequently found as the determinant of maternal health service utilization, contraceptive use or MMR in this review. However, most studies just included educational level in multivariate analyses without a serious discussion of its role in health inequity, perhaps because education level is closely correlated to economic status.

Ethnic minorities, who make up nearly 10% of the population in China, continue to have a lower rate of institutional delivery and pre-natal care utilization than Han ethnicity, placing them at greater risk of maternal mortality. From the included literature, we were unable to determine if the differences in maternal health among women with different educational levels or ethnic background are narrowing or widening in the last decade.

Some studies in several big cities confirmed that rural-to-urban migrants use maternal health care and contraception to a lower extent than permanent residents of cities, and differences in MMR between migrants and permanent residents in the same cities have not shown any narrowing trend.

Gender inequity, which still exists in some areas of China, also influences the utilization of maternal health care and contraceptives and negatively affects maternal health.

The inequity in another two MDG 5 indicators–adolescent birth and unmet need for family planning – was seldom studied by the included literature.

### Intermediary determinants

Some of the included studies also qualitatively explored or discussed the causes for the observed inequities, which were grouped into different kinds of intermediary determinants in the CSDH framework. Some of these intermediary factors were frequently discussed and widely known: long distance to health facilities for the poorest and ethnic minorities’ women who usually living in remote areas; insufficient knowledge on importance of maternal health care and contraceptives among women with low economic status or education; limited knowledge and traditional beliefs resulting in low maternal health care seeking among ethnic minorities; out-of-pocket payments posing financial barriers for health care seeking for poor women; and the poor quality of maternal health services provided by health facilities in rural and remote areas.

Other intermediary factors are less discussed, but are meaningful and can also provide good entry points for policy interventions. For rural-to-urban migrants, who must accept low-paying work and lack health insurance or access to other public-funded programs, there is also greater psychosocial stress when seeking maternal health care because of being not familiar with urban environments and hospitals’ services procedures. Low educational status is related to less capacity to access health information and poor communication with health workers, which restrains low educated women's health care seeking. Lack of privacy and lack of female health workers were discussed by several studies to be barriers of maternal and reproductive health care utilization, especially for ethnic minorities and rural-to-urban migrants. Son preference in Chinese culture and family planning policy interact with each other and are both impeding factors of maternal health: son preference reduces maternal health care utilization for women who already have a son. This influence is intensified by the family planning policy in China because of the financial penalty for unauthorized pregnancy; under the birth planning policy, women and families who have not had a son but with strong son preference may manage to bear a son by some strategies like sex-selective abortion of female fetuses.

Although the CSDH framework includes health systems factors within Intermediary factors, health system factors can influence other intermediary factors: out-of-pocket payments for health care may reduce the affordability of poor populations to access healthy food and clothing, NCMS's reimbursement and subsidy programs can prevent the deterioration of material circumstances, all of which will then impact on maternal health status. However, these interactions and how they finally influence maternal health were not discussed in the included primary studies.

### Relationship to other studies and reviews

We did not find any other reviews synthesizing the different determinants of inequity in maternal health in China. The included primary studies showed very similar research outcomes: whether they were national analysis or small studies at the provincial or regional level, they all concluded that women with low income status, women living in rural or remote areas, less educated women, ethnic minorities and rural-to-urban migrants are still disadvantaged groups in maternal health services utilization or maternal health outcomes. However, the causes of these inequities discussed by included studies were different, and no study covered all four kinds of intermediary factors explaining how inequities were generated.

A systematic review conducted by Say (22) found that in most developing countries, wealthier and urban women were more likely to deliver with the help of a skilled health worker or in medical settings. Say's review quantitatively synthesized urban–rural and economic inequities in the use of maternal health-care in developing countries, but did not systematically review the reasons for these differences. Studies in Viet Nam (68) also found that low-income populations, those living in remote areas, the less educated, ethnic minorities and the low status of women were structural factors relating to poor maternal health. Out-of-pockets payment for maternal health care, long distance to health facilities, limited knowledge of maternal health and poor quality of maternal health services in less-developed areas were important intermediary factors generating inequity. Son preference was also found as a reason of inequity in maternal health care utilization in other patrilineal societies (69, 70), but in China the influence of son preference on maternal health is much more complex because of the family planning policy (66). Just like rural-to-urban migrants of China, studies conducted in other countries (71–73) showed that their migrants also had lower awareness of reproductive health, more high-risk behaviors with regard to reproductive health and low health insurance coverage.

### Study limitations

We did not exclude studies on the basis of methodological quality. This approach was taken for two reasons: firstly because the aim of our literature review is simply to map the overall situation and causes of inequity in MDG 5 in China but not to estimate the extent of inequity. Secondly, some structural or intermediary determinants that we extracted were only discussed by authors but not quantitatively or qualitatively verified, as this would require high methodological quality of studies.

Although we searched four main databases covering journal articles, dissertations and reports on health or specific reproductive health areas, the search of our literature review was not as comprehensive as a systematic review might be. Other relevant grey literature may not have been retrieved, but we think we have covered the main situations and causes of inequity in maternal health in China as expressed in the literature. There may be other causes and explanations, however, these have not been explored by researchers in the published literature such as discrimination.

### Implications for research

This literature review finds some research gaps. We did not find any studies applying the theoretical framework to explore or discuss the causes of the inequities in maternal health. Most of the included studies incorporated all structural and intermediary factors in a multivariate analysis model without any hierarchical approach and cannot quantitatively verify how the structural determinants influence maternal health by pathways of intermediary determinants. Although there are studies showing changes in inequity among economic status groups and between migrants and permanent residents, the longitudinal analyses on inequity in maternal health outcomes among education level groups and between ethnic minorities and Han ethnicity are still lacking. In addition, studies about inequity in adolescent birth and unmet need for family planning are very limited. Also, there is a lack of studies investigating how the relationship between education and maternal health is mediated.

A general problem in all quantitative studies about equity in maternal health in China included is that the total number of those asked to participate in the survey and the data collection tools was not clearly reported in papers. Of the main problems with the quality of included qualitative studies was the lack of justification for qualitative design and detailed description of the research process.

Consequently, in order to find out currently who are disadvantaged populations in maternal health in China, and to fully understand the causes of these inequities and observe if these inequities are improving or worsening, stratified or longitudinal analyses are needed, in addition to more qualitative research on inequity in adolescent birth rate or unmet need for family planning.

### Implications for health policy

Inequity in maternal health continues to be an issue worthy of greater programmatic and monitoring efforts in China. First, government information systems need to be improved to include health indicators for the known disadvantaged groups in order to observe trends of health inequity for evidence-based policy making. Second, central and provincial governments need to implement policies and programs that will decrease health inequities. By grouping the determinants of maternal health into structural and intermediary factors based on the CSDH framework, we can identify some potential policy interventions that may reduce such inequities. For example, long distance to health facilities was frequently mentioned as barrier to accessing health services faced by populations living in rural or remote areas. Out-of-pocket payments also affected certain population's use of maternal health services. This implies that expanded coverage of qualified maternal health care provision in rural or remote areas, and increased subsidies targeting maternal health care in the poorest populations, ethnic minorities and rural-to-urban migrants may be solutions to reducing inequities in maternal health. However specific recommendations to reduce inequity in maternal health will need to be based on systematic reviews of the effectiveness of these interventions. Furthermore, the contextual factors that create disadvantage also need to be addressed. The Chinese government has prioritized equity in its current 5-year plan, which aims at reforming different aspects of socio-economic and public policies, such as the social security system, and infrastructure construction in rural areas. But the implementation of reform plans and their effectiveness on inequity in maternal health still needs to be monitored and evaluated.

## Conclusions

Lowest income populations, those living in remote areas, low-educated populations, ethnic minorities and rural-to-urban migrants are disadvantaged populations in maternal health in China. Gender inequity also contributes to disadvantaged position of women in health. The inequities in maternal health in terms of income level and place of residence are narrowing, but the equity between migrants and permanent residents has not shown any narrowing trend. These inequities are mediated and explained through different kinds of material, behavioral or health system factors.

## Authorship

Beibei Yuan and Sarah Thomsen conceptualized the study. Beibei Yuan conducted the review and analysis. All authors contributed to writing and additional articles to be included.
